# Xiphosurid from the Tournaisian (Carboniferous) of Scotland confirms deep origin of Limuloidea

**DOI:** 10.1038/s41598-019-53442-5

**Published:** 2019-11-19

**Authors:** Russell D. C. Bicknell, Stephen Pates

**Affiliations:** 1 Palaeoscience Research Centre, School of Environmental and Rural Science, University of New England, Armidale, New South Wales 2351 Australia; 2 Museum of Comparative Zoology and Department of Organismic and Evolutionary Biology, Harvard University, 26 Oxford Street, Cambridge, MA 02138 USA

**Keywords:** Palaeontology, Taxonomy

## Abstract

Horseshoe crabs are archetypal marine chelicerates with a fossil record extending from the Lower Ordovician to today. The major horseshoe crab groups are thought to have arisen in the middle to late Palaeozoic. Here we present the oldest known limuloid from the lower Carboniferous (Tournaisian stage, c. 350 million years ago) of Scotland: *Albalimulus bottoni* gen. et sp. nov. A comprehensive phylogenetic analysis supports the placement of *A. bottoni* as a representative of the extant family Limulidae and 100 million years older than any other limulid taxon. The use of geometric morphometric analyses corroborate the erection of the new taxon and illustrates the exploitation of morphospace by xiphosurids. This new taxon highlights the complex evolutionary history of xiphosurids and the importance of documenting these unique Palaeozoic individuals.

## Introduction

Horseshoe crabs have a highly conservative and iconic shape: a crescentic prosomal shield, opisthosomal tergites fused into a thoracetron, and a styliform telson^1,2^. The documentation of fossil and extant^3–10^ representatives of true horseshoe crabs (Xiphosurida) started in the early 1800’s^11,12^ and has continued to this day: a research effort resulting in 80 fossil species^13^. However, this number continues to change as a better understanding of the taphonomic and ontogenetic variation of taxa allows identification of invalid species^14,15^. Recent phylogenetic works^16–18^ have augmented this research and presented a more complete understanding of the evolutionary history of Xiphosurida. Although horseshoe crabs have a fossil record extending from the Lower Ordovician (c. 480 million years ago) to the present day^19,20^, Xiphosurida arose close to the base of the Carboniferous^21^. After this origin, horseshoe crabs diversified and three of the five xiphosurid groups (families) arose: Belinuridae, Paleolimulidae, and Rolfeiidae. Carboniferous belinurids were the most abundant group at this time^14,22–25^ with 37 different species arrayed across the genera *Alanops* Racheboeuf, Vannier & Anderson, 2002^26^, *Bellinurus* Pictet, 1846^27^, *Euproops* Meek, 1867^28^, *Liomesaspis* Raymond, 1944^23^, *Prolimulus* Frič, 1899^24^, and *Stilpnocephalus* Selden, Simonetto & Marsiglio, 2019^25^. The four Carboniferous paleolimulid species^29–32^ are arrayed across *Paleolimulus* Dunbar, 1923^33^ and *Xaniopyramis* Siveter and Selden, 1987^34^. Finally, Rolfeiidae is a monospecific group containing *Rolfeia fouldenensis* Waterston, 1985^35^. Furthermore, the Superfamily Limuloidea—the group containing Limulidae and Austrolimulidae—is represented in the Carboniferous by *Valloisella lievinensis* Racheboeuf^36^. Limulidae, the group of horseshoe crabs containing all extant species, first appears in the Triassic, c. 100 million years later^37,38^. Given that Limuloidea has a Carboniferous presence, it would be logical to suggest that limulids may also have a deeper origin than previously thought. Here we present a new Carboniferous-aged limuloid from the south of Scotland, *Albalimulus bottoni* gen. et sp. nov. The morphology of this animal suggests a likely limulid affinity and a comprehensive phylogenetic analysis places this taxon within Limulidae. The identification of this taxon suggests that crown group horseshoe crabs potentially arose much earlier than previously thought: just above the Devonian-Carboniferous boundary (Tournaisian stage).

## Specimen History and Geological Setting

**Figure 1:**
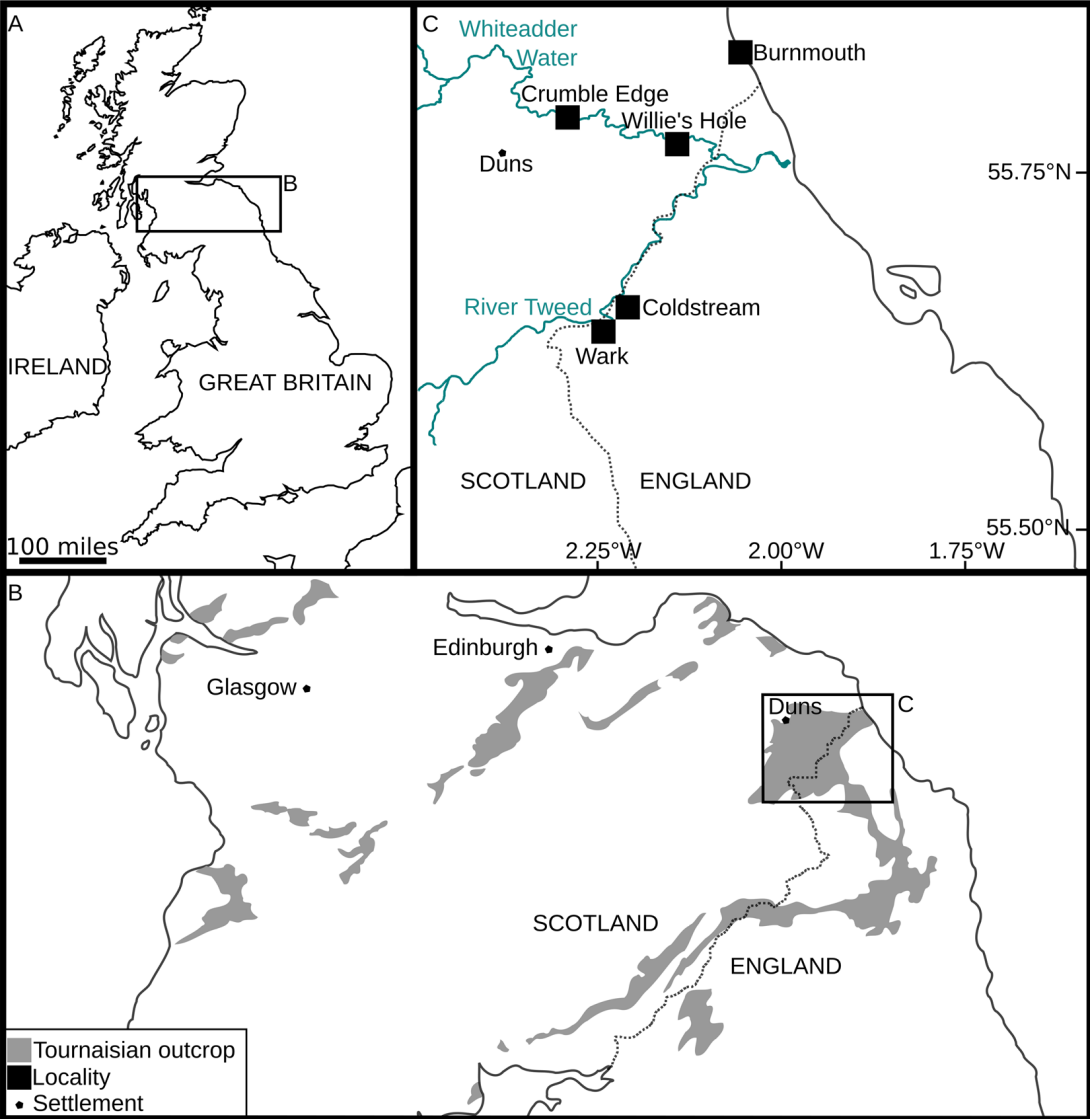
Map showing Tournaisian outcrop and localities close to Duns. (**A**) Outline of Great Britain. (**B**) Rectangle in (**A**), map of northern England, showing Tournaisian outcrop. Redrawn from Smithson, *et al*.^40^ with location of Duns identified from Google Maps. Based on BGS Materials © UKRI. (**C**) Rectangle in (**B**), map of area surrounding Duns, showing nearby localities. Redrawn using Inkscape. Figures used are from Kearsey, *et al*.^39^ and Smithson, *et al*.^40^ with location of Duns and river paths added using Google Maps (Map data ©2019 Google). Maps contain Ordnance Survey data © 1028 Crown copyright and database right 2016.

The historical nature of the material (collection year unknown) means that limited information is available regarding its geological and locality setting. The British Geological Survey (Keyworth) specimens, representing a part and counterpart, were collected from a river section of the lower Calciferous Sandstone Series at Whiteadder Water. Unfortunately, the exact horizon in this section is unknown due to lack of collection data. The recorded coordinates of the specimens (55.797878°N, 2.277510°W) give a position slightly east of Duns (Berwickshire, Scotland). The locality known as Crumble Edge, highlighted in the recent literature^39,40^ (Fig. 1), is along the Whiteadder Water, and has coordinates nearly identical to those reported in the British Geological Survey data (see coordinates of Crumble Edge taken from Kearsey, *et al*.^39^, their Fig. 1). We therefore suggest that the British Geological Survey specimen was collected from, or close to, Crumble Edge.

**Figure 2:**
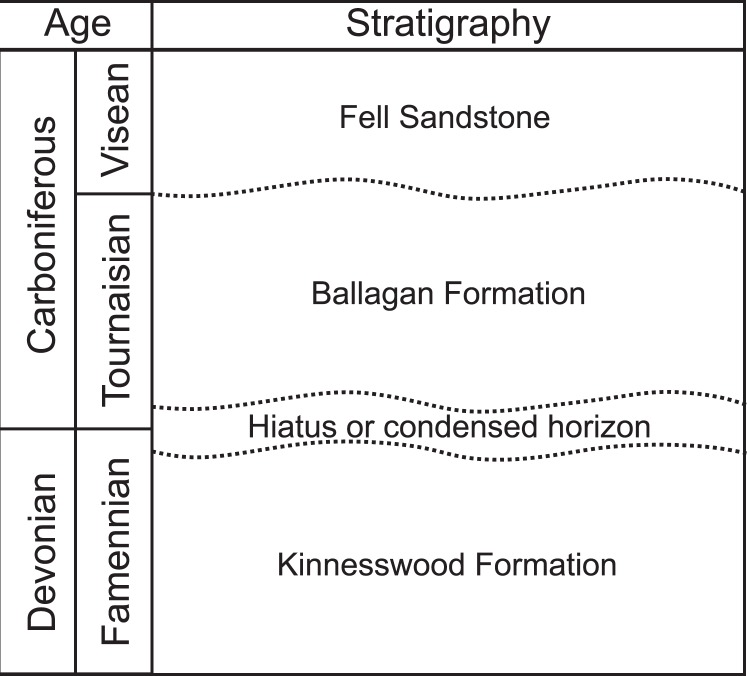
Upper Devonian and lower Carboniferous stratigraphy of the Scottish Borders. Redrawn from Marshall, *et al*.^41^.

The Ballagan Formation outcrops across the Midland Valley of Scotland and northern England^39^ (Fig. 1), where it was previously known as the lower part of the Calciferous Sandstone Series and Cementstone Group respectively. The Ballagan Formation extends through the entire Tournaisian (lower Carboniferous), with the Devonian-Carboniferous boundary positioned close to the top of the underlying Kinnesswood Formation^41^ (Fig. 2). Recent studies exploring the first terrestrialisation of tetrapods have furthered the understanding of the sedimentology, palynology, stratigraphy, and palaeoenvironment of the Ballagan Formation, which consists of a thick succession of red and grey siltstones, fine sandstones, ferroan dolostones, and over 200 separate palaeosol horizons^39,40,42–44^ (Figs 1, 2). These likely record a seasonal climate with a mosaic of closely packed distinct habitats from both coastal floodplain and marginal marine environments^39^. The fauna is a diverse group of bivalves, ostracods, eumalacostracans, spinicaudatans, scorpions, millipedes, and tetrapods^43–46^.

Crumble Edge is a 46 m thick succession exposed in a river cliff near Duns, close to the base of the Ballagan Formation^39^. It is therefore close to the Devonian-Carboniferous boundary, although its exact correlation with other, more extensive Ballagan Formation outcrops is unknown. It contains 18 palaeosols (mostly Inceptisol) and has been recently logged at high resolution^39^. A small amount of tetrapod material has been reported from this site^40^; however, the majority of the recent studies considering tetrapods or invertebrates have used material from other nearby sites^42–45^(Fig. 1).

## Materials and Methods

The single known specimen is housed within the British Geological Survey (BGS.GSE), Keyworth, where it is curated under two different catalogue numbers for the part (BGS.GSE2028) and counterpart (BGS.GSE9680). BGS.GSE2028/9680 is preserved as a flat impression on a small slab of clayey, grey-black shale. When describing this specimen, we followed the systematic taxonomy^16,17^ and the anatomical terminology^5,38,47^ of previous workers. The specimens were coated with ammonium chloride sublimate and photographed under LED lighting using a Canon EOS 5DS digital camera and a Canon MP-E 65 mm 1–5x macro lens housed at the University of New England. Images were stacked using Helicon Focus 7 (Helicon Soft Limited) software.

**Figure 3:**
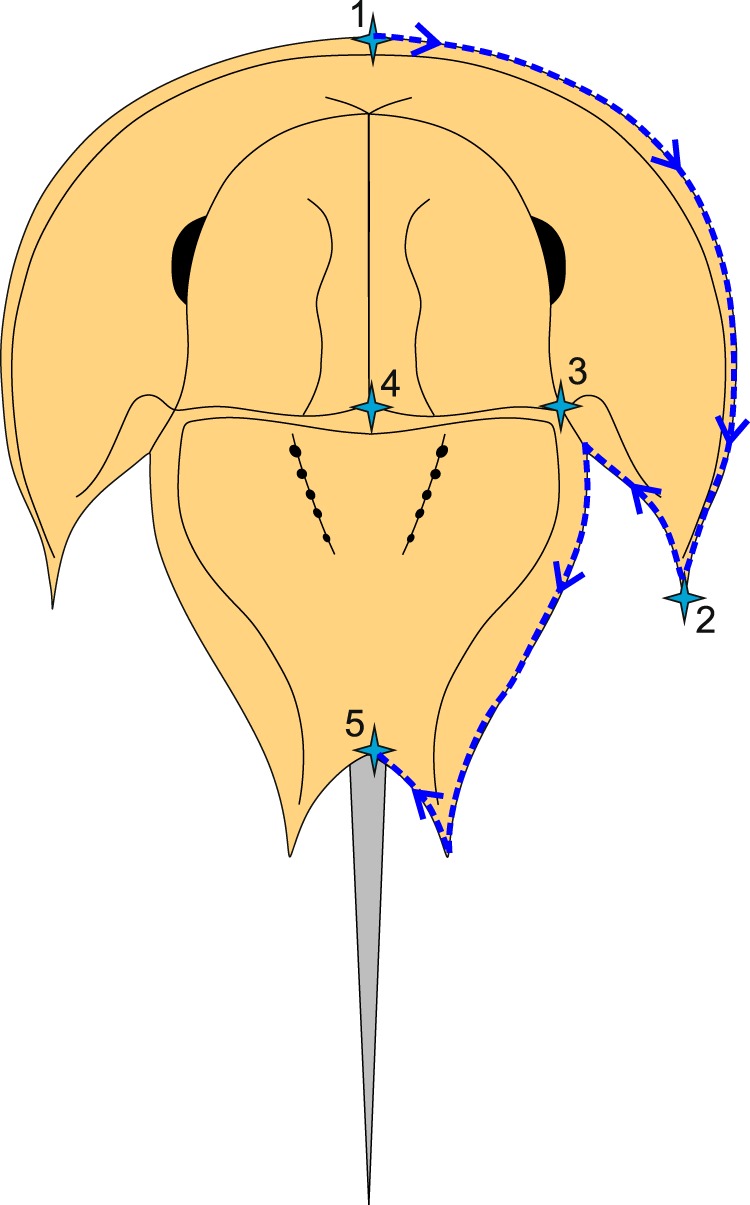
Approximate semilandmark trajectory (blue arrows and dotted line) and the five digitised landmarks used here. Landmarks are described in Table 1.

Following Bicknell, *et al*.^38^, a morphometric analysis using landmarks and semilandmarks of 82 specimens was conducted to quantitatively assess the morphology of BGS.GSE2028/9680 relative to other taxa. The examined species were from Austrolimulidae, Belinuridae, Limulidae, Paleolimulidae, Rolfeiidae, and stem xiphosurids (*sensu* Bicknell, *et al*.^21^). Landmarking and semilandmarking was conducted using the Thin-Plate Spline (TPS) suite (http://life.bio.sunysb.edu/morph/index.html). A TPS file was constructed using tpsUtil64 (v.1.7). The TPS file was imported into tspDig2 (v.2.26), which was used to place five landmarks and 50 semi-landmarks along the right prosomal shield and thoracetron (Fig. 3; Table 1). Semilandmarks were placed in a clockwise direction along the most anterior section of the prosomal shield, ending in the most posterior section of the thoracetron. Points were digitised as *xy* coordinates. The thoracetron was digitised along the right thoracetronic margin, but excluding areas containing moveable and fixed spines, as they are not known to, or preserved in, all horseshoe crab taxa. In cases where the right side was poorly preserved, the left side was used instead, and the data mirrored. These points populated the TPS file with landmark and semilandmark data (Supplementary Information 1). The TPS file was imported into an R environment. The ‘geomorph’ package^48^ was used to conduct a Procrustes Superimposition and Principal Components Analysis (PCA) of the data. Procrustes Superimposition standardises for size and orientation such that only shape variation was assessed (Supplementary Data 2). The PC data were output and logged in Supplementary Data 3. Only the first two Principal Components (PCs) were considered as they explained the majority of the variation in the data (67.8%).

**Table 1:** Description of landmarks digitised for the geometric morphometric analysis depicted in Fig. 3.

Landmark number	Description of landmark
Landmark 1	Most anterior point on the sagittal line of the prosomal shield
Landmark 2	Most distal point along genal spine
Landmark 3	Most posterior point along ophthalmic ridge
Landmark 4	Most distal point along sagittal line of prosomal shield
Landmark 5	Most distal point of the thoracetron along the sagittal line. Corresponds to thoracetron-telson joint

To evaluate the phylogenetic position of *Albalimulus bottoni* gen. et sp. nov., we coded it as an additional taxon in the recently published matrix of Lamsdell^17^; a matrix that contains a broad sampling fossil and extant euchelicerates (Supplementary Information 4). The analysis was performed under equal-weights parsimony in TNT 1.5^49^ utilising the “New Technology” tree search strategy using random sectorial searches, 1000 iterations of the parsimony ratchet, 50 cycles of drifting and 5 rounds of tree fusing. All multistate characters were considered unordered as in the original analysis.

A further matrix was compiled to align with the morphometric analyses and to explore the impact of involving more than one additional taxon in this matrix. Ten additional taxa were therefore coded into the Lamsdell^17^ matrix (Supplementary Information 5). This matrix was analysed using the same parameters as the Supplementary Information 4. The maximum parsimony tree produced a large polytomy that collapsed Paleolimulidae, Austrolimulidae and parts of Limulidae. This is not informative for uncovering evolutionary relationships so was not considered at length here.

### Systematic palaeontology

Euchelicerata Weygoldt and Paulus, 1979^50^

Order Xiphosurida Latreille, 1802^4^

Suborder Limulina Richter and Richter, 1929^51^

Superfamily Limuloidea Zittel, 1885^52^

Family Limulidae? Zittel, 1885^52^

Genus *Albalimulus* nov. gen.

#### Etymology

*Albalimulus* is a combination of the Gaelic name for Great Britain (*Alba*) and *Limulus*, the genus of the extant and iconic North American horseshoe crab and commonly used suffix in generic names of representatives of Limuloidea.

#### Type species

*Albalimulus bottoni*, new species.

#### Distribution

Ballagan Formation, Tournaisian, lower Carboniferous (Figs. 1, 2).

#### Diagnosis

As for species.

*Albalimulus bottoni* nov. sp.

Figures 4, 5

**Figure 4:**
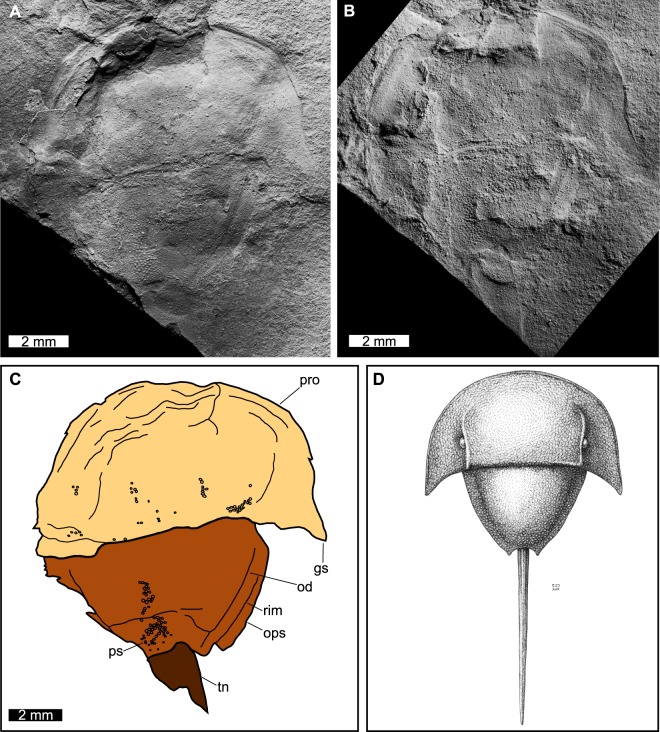
*Albalimulus bottoni* gen. et sp. nov. holotype BGS.GSE2028/9680. (**A**) Complete specimen of BGS,GSE2028 (part). (**B**) Complete specimen of BGS.GSE9680 (counter-part). Image mirrored to align with (**A**). Both specimens were coated with ammonium chloride sublimate and converted to grey-scale. (**C**) Line drawing of BGS.GSE2028 highlighting important anatomical features. The left prosomal side is preserved less completely than the right side. (**D**) Idealised reconstruction of *A. bottoni*. Note that the telson is styliform, reflecting extant taxa; however, the degree of telson elongation cannot be confirmed from the holotype. Abbreviations: gs, genal spine; od, opisthosomal doublure; ops, opisthosoma; pro, prosoma; ps, pustulose cuticle; rim, opisthosomal rim; tn, telson. Image credit (**D**): Elissa Johnson.

**Figure 5:**
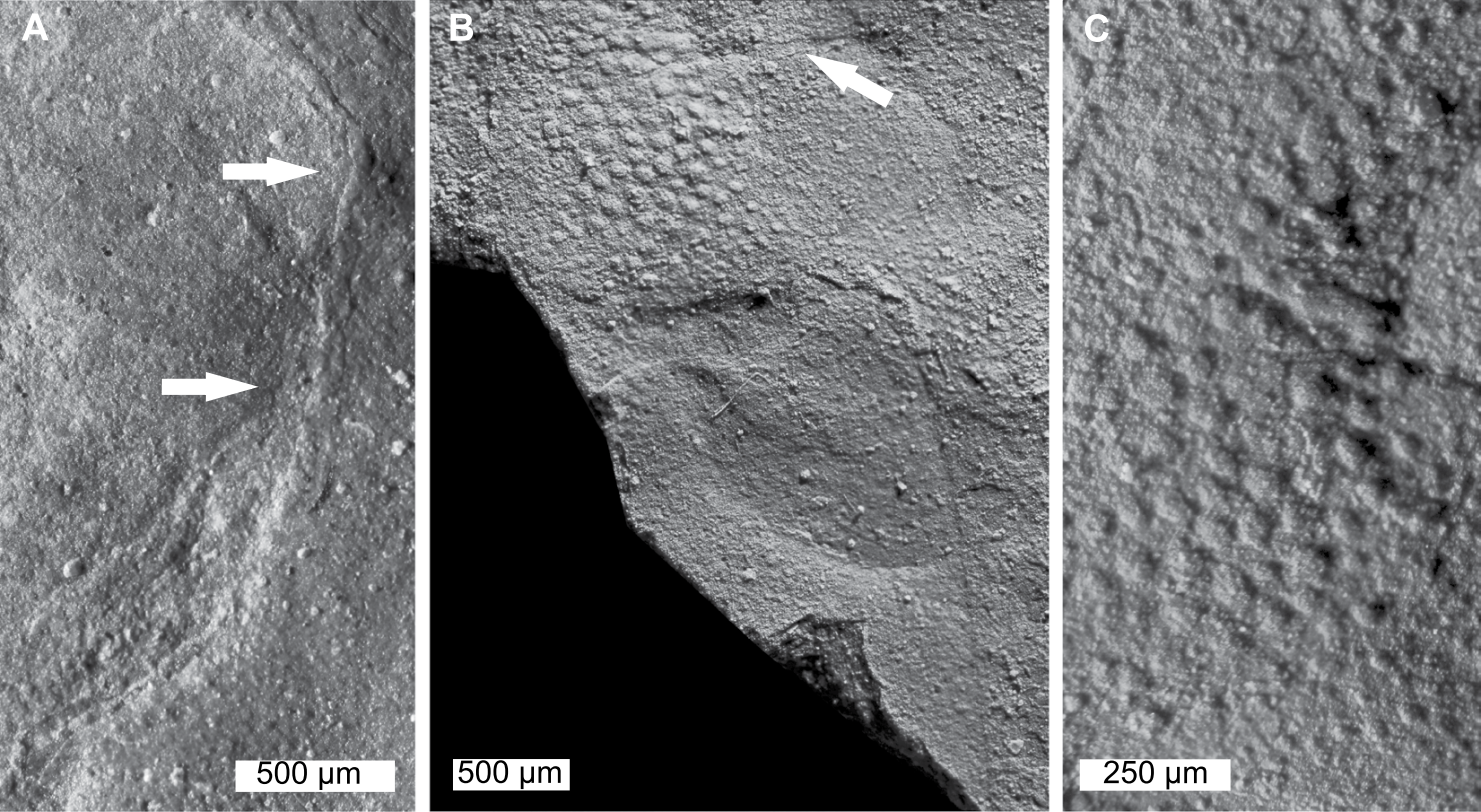
Close up of informative and unique features of *Albalimulus bottoni* gen. et sp. nov. (**A**) Right ophthalmic ridge (white arrows). (**B**) Thoracetron-telson joint showing pustules and linear structure (white arrow). (**C**) Pustulose cuticle ornamentation on thoracetron. Specimens were coated with ammonium chloride sublimate and converted to grey-scale.

#### Etymology

Trivial name *bottoni* was chosen in recognition of Mark L. Botton who has contributed extensively to extant horseshoe crab research and conservation of *Limulus polyphemus* (Linnaeus, 1758)^53^ across his career.

#### Holotype by monotypy

BGS.GSE2028/9680 (part/counterpart).

#### Distribution

Same as for genus.

#### Type locality and horizon

The Whiteadder Water river, near Duns, Berwickshire, Scotland (55.797878°N, 2.277510°W), likely at or very close to Crumble Edge locality of Kearsey, *et al*.^39^ and Smithson, *et al*.^40^ (Figs 1, 2).

#### Diagnosis

Limuloid with pustulose cuticular ornament, most prominent on the thoracetron; well-defined, curved prosomal-opisthosomal hinge; prosomal shield is slightly longer than thoracetron.

#### Preservation

BGS.GSE2028/9680 is preserved flattened as part and counterpart on a thin slab of siltstone.

#### Description

BGS.GSE2028/9680 is an articulated prosomal shield, thoracetron, and partial telson preserved as a part and counterpart (Figure 4A–C). Almost no relief is observed. Specimen is 12.5 mm long, including the preserved telson section. Prosoma is semi-circular and 5.4 mm long sagittally. The right side of the prosoma is preserved better than the left side. Prosomal width across the posterior margin of lateral rims is 10.2 mm. A thin prosomal rim is preserved along margins and attains a greatest width of ca. 0.5 mm. No prosomal doublure is visible. Left ophthalmic ridge is preserved as a slight impression that curves out towards the left lateral border. The left ophthalmic ridge is 2.4 mm long and the anterior section is slightly obscured by rock. The right ophthalmic ridge is preserved as a slight impression and curves out towards the right lateral border (Fig. 5A). The right ophthalmic ridge is 2.8 mm long. No lateral compound eyes can be confidently discerned. No cardiac lobe or associated ridges are noted (Fig. 4A). Ocelli are not observed. The left genal spine is not preserved. The right genal spine is completely preserved, 2 mm long and extends posteriorly to 15% of the thoracetron length. The genal spine tip is 6.9 mm from the prosomal midline. The lateral extent between the right genal spine tip and thoracetron is 2.9 mm. Angle between the right genal spine and right side of the thoracetron is 80°. Prosomal-opisthosomal hinge is pronounced, 5.6 mm wide and 0.3 mm long. The hinge curves posteriorly towards the lateral sides of BSG.GSE 2028/9680. The posterior right section of prosoma has pustulose cuticular ornament. No prosomal appendages are preserved.

The thoracetron is trapezoidal, 4.6 mm long and 7.8 mm wide anteriorly, tapering to 2.3 mm posteriorly. The left side is preserved less completely than right side. No axial lobes are noted. No apodemal pits are noted. No definitive evidence for tergal expression is noted. A potential opisthosomal doublure is noted. Doublure is 6.6 mm anteriorly tapering to 3.5 mm posterior (these are minimum values as outer-most section of the left side of the doublure is not preserved). This feature may also reflect compression through preservation. A thin thoracetronic rim is noted on the right side of the thoracetron and only slightly pronounced. The rim is 0.8 mm wide anteriorly tapering to 0.5 mm posteriorly. No fixed or moveable spines are noted. Left side of thoracetron has pustulose cuticular ornament preserved (Fig. 5B,C).

The telson is partly preserved and is articulated with the posterior thoracetronic margin (Fig. 5B). Margin between the thoracetron and telson curves slightly towards the anterior of the specimen. Telson is partly preserved, is 2.8 mm long and 1.9 mm wide anteriorly (minimum values). Telson lacks an axial ridge.

#### Remarks

*Albalimulus bottoni* preserves select characters diagnostic of at least Limuloidea: the horseshoe shaped prosoma and trapezoidal thoracetron, non-converging ophthalmic ridges (*sensu* discussion in Lamsdell^17^). Other diagnostic and informative features such as appendages, moveable spines, dorsal keel, and compound eyes are not present or preserved. The combined presence of pustulose cuticle, possible lack of pronounced apodemal pits, and pronounced prosomal-opisthosomal hinge are unique and warrant the erection of a new genus and species (Fig. 4D). This outcome is corroborated by the placement of this taxon in a unique position in xiphosurid morphospace (see Geometric Morphometric analysis below).

Belinurina, which includes the Belinuridae, is characterised by the possession of pronounced, concave ophthalmic ridges meeting anteriorly, the presence of ophthalmic spines, a pronounced axial region exhibiting clear segmentation, a marked posterior opisthosomal boss likely associated with powerful telsonic musculature, and a thoracetron bordered by either incised marginal spines, or a flat marginal flange resulting from the fusion of the base of these marginal spines. The general outline of the thoracetron is roughly semi-circular. Since *Albalimulus bottoni* does not exhibit any of these characters, it can be confidently excluded from Belinurina.

The overall morphology of *Albalimulus bottoni*, showing a trapezoidal outline of the thoracetron, is strongly suggestive of an affinity within the Suborder Limulina. However, *A. bottoni* lacks a clearly expressed segmented axis and pyramidal cheek nodes, interophthalmic ridges, and prosomal transverse ridge nodes that characterise Paleolimulidae, with its effaced morphology is indicative of Limuloidea. The apparent lack of fixed or moveable marginal spines is shared with Austrolimulidae and *Valloisella lievinensis*; however, this feature may be absent due to the preservational mode. More specimens are therefore needed to confirm whether the lack of potentially diagnostic features is biological or taphonomic. Regardless, *A. bottoni* lacks the elongated posterior pleurae forming an embayment surrounding the base of the telson and outwardly directed genal spines, typical of the Austrolimulidae. The results of the phylogenetic analysis (discussed in Phylogenetic Results) favour the placement of *A. bottoni* in a basal position within Limulidae. However, we are tentative in this placement, due to the singular nature of BGS.GSE 2028/9680, the preservation precluding the identification of all diagnostic characters of the Family, and the poor resolution of phylogenetic analyses involving more taxa (Supplementary Data 5).

A consideration must be given to the linear structure on the posterior section of the thoracetron (Fig. 5B). This lineation is 3.2 mm long and located 2.0 mm posteriorly from the prosomal-opisthosomal hinge. We are unconvinced that it represents a tergite, especially as one would expect to observe at least two structures. We suggest it reflects compression of the fossil, an interpretation supported by the uneven outline. A tergal boundary would be smooth; however, the effaced right side of the fossil may be the reason why the apparent boundary cannot be identified across the entire fossil. Regardless, this potential tergal boundary is not pronounced enough to represent an articulation, and so was unlikely functional. Nonetheless, the presence of tergites would not affect the placement of *Albalimulus bottoni* in Limuloidea as the group contains taxa that have tergal expression: the austrolimulid *Austrolimulus fletcheri* Riek, 1955^54^ and the limuloid *Valloisella lievinensis*^55^. It does raise doubts about a limulid affinity.

One of the key autapomorphies of *Albalimulus bottoni* is the pustulose cuticle that is unknown to Xiphosura or Xiphosurida. The feature has been documented in related euchelicerates: arachnids^56^, chasmataspidids^57^, and eurypterids^58,59^, and is also present in other arthropod clades, e.g. aglaspidids and cheloniellids. The pustulose ornament unlikely reflects cuticular secretion, as extant taxa would also commonly exhibit these features^60^. It has been hypothesised that pustulose ornament potentially provided additional sensory capability and camouflage for benthic euchelicerates^61^. *Albalimulus bottoni* would have benefited from additional camouflage as it was so small relative to contemporary predators. Research into pustulose ornament in decapod crustaceans may uncover a functional use for this feature in arthropods.

## Morphometric Results

**Figure 6:**
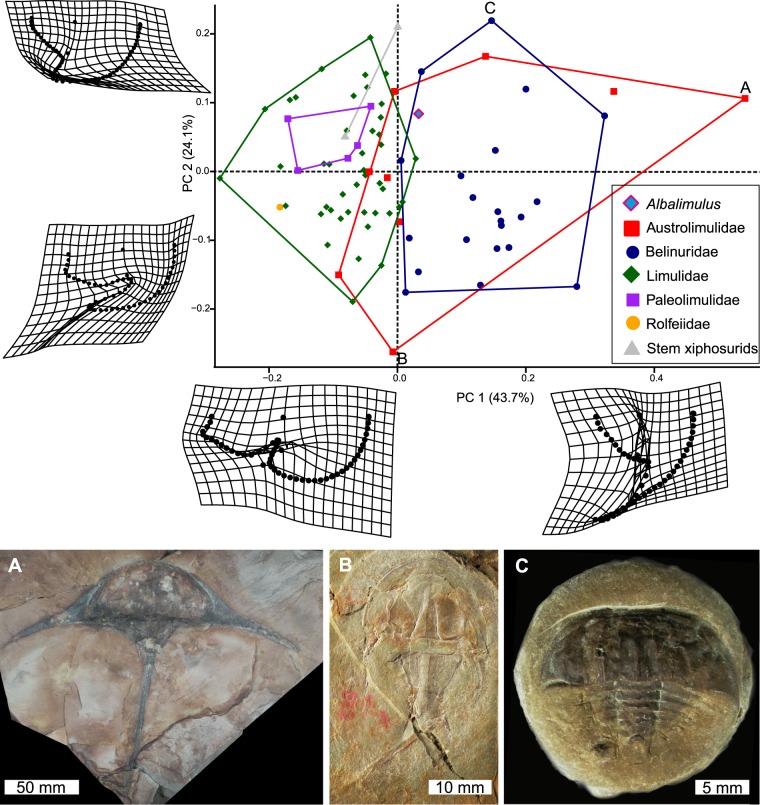
Xiphosurids in Principal Component space. The thin plate spline reconstructions represent factor loadings for PCs 1 and 2. They also demonstrate the extreme morphological variation along these PCs. Austrolimulids and belinurids fall in positive PC1 space; reflecting their extreme genal spine morphologies. Limulids and paleolimulids occupy more negative PC1 space. *Albalimulus bottoni* falls into the extremes of shape space occupied by Belinuridae and Austrolimulidae, in an area that almost overlays Limulidae. (**A**) *Austrolimulus fletcheri* (Australian Museum specimen AM F 38274, holotype). Note the large genal spine splay. (**B**) *Psammolimulus gottingensis* (Geowissenschaftliches Zentrum der Georg-August-Universität Geowissenschaftliches Museum specimen GZG INV 15356a 10). Note the indentation of the proximal genal spine section into the prosomal shield. (**C**) *Bellinurus trilobitoides* (Natural History Museum UK specimen NHMUK IP In 59324, holotype). Photo credit (**A**): Josh White; (**B**): Gerhard Hundertmark.

**Figure 7:**
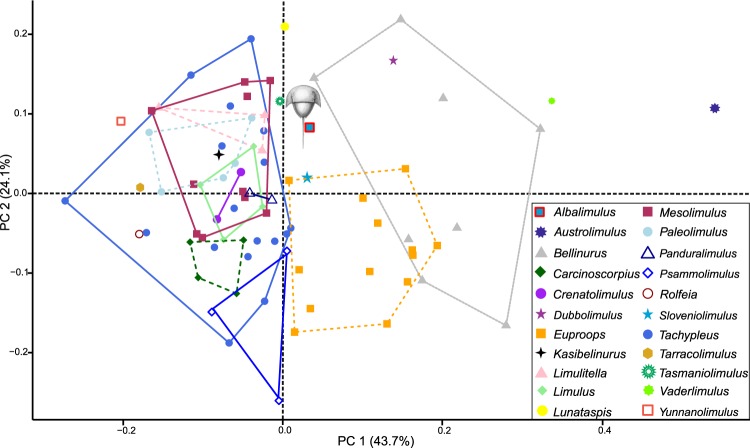
Principal Component plot detailing morphospace space occupied by genera. Taxa are bound by convex hulls. *Albalimulus bottoni* falls outside all convex hulls, which demonstrates that it represents a new taxon.

The PCA plots demonstrate Family and generic distribution in morphospace. PC1 describes how laterally extended the genal spine tip is relative to prosoma and whether the thoracetron has a trapezoidal or round shape (Fig. 6). Austrolimulids (Fig. 6A,B) and belinurids dominate positive PC1 space as most of these taxa have large, laterally and posteriorly extended genal spines. Negative PC1 space is dominated primarily by limulids and paleolimulids: taxa with less accentuated genal spines. Notably, a selection of proposed austrolimulids (*sensu* Lerner, *et al*.^47^ and Bicknell^62^) are located in more negative PC1 space—these taxa have a less pronounced genal spine splay (Fig. 6B). PC2 (24.1% shape variation) describes the degree to which the proximal section of the genal spine is indented into the prosomal shield. *Psammolimulus gottingensis* Lange, 1923^63^ has the most negative PC2 value as the proximal genal spine section is highly indented (Fig. 6B). Conversely, *Bellinurus trilobitoides* (Buckland, 1837)^64^ is located in positive PC2 space as the genal spines are not indented into the prosomal shield (Fig. 6C). *Albalimulus bottoni* is located close to the origins of both PC axes (PC1 = 0.03, PC2 = 0.08; Figs 6, 7). The genus is located within the outer limits of the Belinuridae and Austrolimulidae convex hulls, at a place in PC space that almost overlays the Limulidae convex hull (Fig. 6). The generic distribution of specimens shows that *A. bottoni* is not bound by any convex hulls (Fig. 7).

## Phylogenetic Results

**Figure 8:**
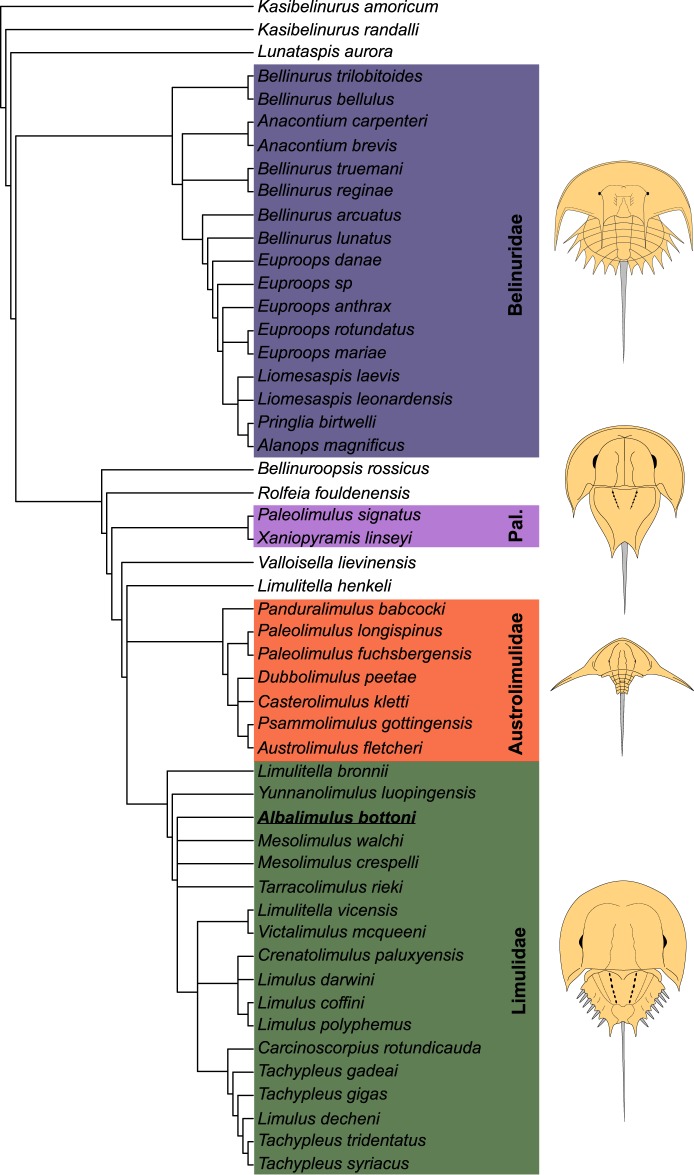
Xiphosurid phylogeny produced using the Lamsdell^17^ phylogenetic matrix. *Albalimulus bottoni* (in bold and underlined) is located close to the base of Limulidae. Major groups are colour coded to match Fig. 5. Other clades coded into this phylogenetic matrix (Supplemental Data 4) were not presented as their placement in the phylogeny is unchanged and they do not relate directly to the current research. Abbreviation: Pal., Paleolimulidae.

A phylogenetic analysis resulted in nine most parsimonious trees (CI: 0.468, RI: 0.877, tree length 743). The overall topology of a strict consensus tree produced from these trees (Fig. 8) reflects previous phylogenies that used the same matrix (see^17,18,21,62^). Our phylogenetic analysis placed *Albalimulus bottoni* within Limulidae, in a polytomy close to the base of the group. This polytomy contains *Tarracolimulus rieki* Romero and Via Boada, 1977^65^, *Mesolimulus crespelli* Vía Boada, 1987^66^, *M. walchi* (Desmarest, 1822)^11^, and the stem leading to extant taxa. Most notably, the node leading to *Mesolimulus* Størmer, 1952^8^ observed in Lamsdell^17^ is collapsed into the polytomy in Fig. 8. A phylogenetic matrix that coded an additional 10 taxa collapsed many established families and resulted in a large polytomy (Supplemental Data 5).

## Discussion

The systematic and phylogenetic placement of *Albalimulus bottoni* indicates that the Limuloidea, and potentially Limulidae, had evolved by the early Carboniferous (Tournaisian). If *A. bottoni* does indeed represent the oldest representative of Limulidae, it demonstrates that four xiphosurid groups had a Carboniferous origin and supports previous theories exploring this idea^17^. This also potentially extends the temporal range of the limulids by around 100 million years from the Triassic^67^ to the Mississippian and ultimately demonstrates that xiphosurid evolution is more complex than previously thought. The major limulid evolutionary events after *A. bottoni* are increased size, aligning with Cope’s Law^68^, and the maintenance of an overall morphology similar to *A. bottoni*. This suggests that evolutionary conservation within the group may have persisted over 350 million years^69^. Curiously, the key autapomorphy of *A. bottoni*—the pustulose cuticle ornament—was not retained, suggesting that it represents a derived trait in this taxon, or was lost in younger taxa.

Bicknell, *et al*.^38^ highlighted that geometric morphometric analyses of horseshoe crabs are strongly influenced by extreme prosomal morphologies; as exemplified by austrolimulids. The placement of morphologies with hypertrophied genal spines in very positive PC1 space reconfirms this observation. The possession of extremely pronounced genal spines is also associated with the habitation of freshwater conditions (explored in Anderson^70^ and more recently in Bicknell^62^). PC1 may therefore record a morphological response to palaeoenvironmental conditions and associated life modes. What then is the advantage of the larger genal spine splay? Fisher^71^ suggested that large genal spines might be used in sub-aerial activity and potentially be used as a defensive feature—an observation that aligns with evidence for predation thereon^72^. Alternatively, the hypertrophied genal spines may have served to reduce the impact of uni-directional currents in freshwater conditions, having a stabilising effect^70^. This thesis can be tested using computational fluid flow—a method that was recently used to model the functional impact of fluid on the horseshoe crab carapace^73^. Extending this method to analysing three-dimensional reconstructions of taxa with overdeveloped genal spine morphologies therefore represents a key direction for uncovering the impact (if any) that genal spines had on water-flow about the dorsal carapace.

As *Albalimulus bottoni* is the oldest known limuloid, and likely the oldest limulid, the life mode of the fossil species can be suggested by comparing to extant taxa^7,74–78^. Extant horseshoe crabs are omnivorous marine organisms that use gnathobases on the walking legs to masticate prey such as small molluscs, crustaceans and polychaete worms^74,75,79^. BGS.GSE 2028/9680 was collected from a succession that includes coastal floodplain, and marginal marine deposits; this indicates that the new taxon was likely marine, with a mode of life similar to that of extant taxa. It may also have experienced an infaunal life mode similar the comparably sized, immature extant individuals, as a detrital feeder^7,80^.

## Conclusion

*Albalimulus bottoni* from the lower Carboniferous (Tournaisian) of Scotland represents the oldest known limuloid. Furthermore, the phylogenetic and geometric morphometric analyses presented here suggest that the taxon is most likely a limulid. These findings highlight that Limuloidea, and potentially Limulidae, had a deeper origin than previously documented and conforms to phylogenetic estimates from previous authors. If the phylogenetic placement of *A. bottoni* in Limulidae is correct, this discovery also shows that four of five xiphosurid groups had an origin in the Carboniferous. Regardless, the new specimen demonstrates that horseshoe crabs had a far more complex and diverse evolutionary history than previously noted and that further work is now needed to fill in the gap between *A. bottoni* and more recent limulids.

## Supplementary information

Supplementary Information

Supplementary Information

Supplementary Information

Supplementary Information

Supplementary Information
